# ADOLESCENT GLUTEN INTAKE: POPULATION-BASED STUDY IN A BRAZILIAN CITY

**DOI:** 10.1590/1984-0462/;2019;37;4;00014

**Published:** 2019-07-04

**Authors:** Daniela de Assumpção, Caroline Dario Capitani, Ana Carolina Rocha, Marilisa Berti de Azevedo Barros, Antonio de Azevedo Barros

**Affiliations:** a Universidade Estadual de Campinas, Campinas, SP, Brazil.

**Keywords:** Adolescent, Glutens, Food consumption, Health surveys, Adolescente, Glúten, Consumo de alimentos, Inquéritos epidemiológicos

## Abstract

**Objective:**

To estimate the prevalence of gluten intake according to demographic, socioeconomic, and health-related behavioral variables in adolescents.

**Methods:**

This is a population-based cross-sectional study with a two-stage cluster sampling, conducted in Campinas, São Paulo, in 2008-2009. Foods containing gluten were identified using a 24-hour Recall. We calculated the prevalence and adjusted prevalence ratios with multiple Poisson regression.

**Results:**

The study had a sample of 924 adolescents aged 10 to 19 years. Among the foods assessed, 26.9% (confidence interval of 95% - 95%CI 25.3-28.6) contained gluten. We found a higher prevalence of gluten intake in younger individuals (10 to 14 years), as well as in subgroups of adolescents who had a higher number of household appliances, attended school, consumed fewer beans and vegetables during the week (<4 times), and whose head of the family had better education level (≥12 years of schooling). The main food sources of gluten in their diet were: bread, cakes, and cereals (30.2%), chocolate milk (14%), chicken nuggets (12.3%), and cookies (11%).

**Conclusions:**

The results of the study show the epidemiological profile associated with gluten intake in adolescents and could support actions aimed at promoting healthy eating habits and preventing gluten-related diseases.

## RESUMO

**Objetivo:**

Estimar a prevalência da ingestão de alimentos com glúten segundo variáveis demográficas, socioeconômicas e de comportamentos relacionados à saúde em adolescentes.

**Métodos:**

Trata-se de estudo transversal de base populacional, com amostra por conglomerados e em dois estágios, realizado em Campinas, São Paulo, em 2008-2009. Os alimentos com glúten foram identificados por meio do Recordatório de 24 horas. Calcularam-se prevalências e razões de prevalência ajustadas por meio de regressão múltipla de Poisson.

**Resultados:**

Participaram do estudo 924 adolescentes de dez a 19 anos. Entre os alimentos referidos, 26,9% (intervalo de confiança de 95% - IC95% 25,3-28,6) continham glúten. Prevalências superiores de ingestão de glúten foram verificadas nos indivíduos mais jovens (dez a 14 anos), bem como nos subgrupos de adolescentes cujo chefe de família era mais escolarizado (≥12 anos de estudo), nos que possuíam maior número de equipamentos domésticos na residência, nos que frequentavam a escola e naqueles que consumiam menos feijão e hortaliças durante a semana (<4 vezes). As principais fontes alimentares de glúten na dieta foram: pães, bolos e cereais (30,2%), achocolatado (14%), *nuggets* (12,3%) e biscoitos (11%).

**Conclusões:**

Os resultados do estudo mostram o perfil epidemiológico associado ao consumo de glúten em adolescentes e podem subsidiar ações voltadas à promoção de hábitos alimentares saudáveis e de prevenção de doenças relacionadas ao glúten.

## INTRODUCTION

Once considered a rare condition, the celiac disease presents diverse clinical manifestations, and its diagnosis depends on the combination of serologic, histological, and clinical findings.^1^ ^,^ ^2^ According to the Clinical Protocol and Therapeutic Guidelines for Celiac Disease from the Ministry of Health, anti-transglutaminase antibody (anti-TTG) - immunoglobulin A (IgA) class -, determined by the Enzyme-Linked ImmunoSorbent Assay (ELISA), is the most effective serological test to screen gluten-intolerant individuals.^2^ Positive serology does not substitute the biopsy of the small intestine for histopathological examination, considered the gold standard test in celiac disease diagnosis.^2^

A potential risk factor associated with the increasing prevalence of celiac disease and other gluten-related disorders, such as dermatitis herpetiformis, wheat allergy, and gluten sensitivity, is the high exposure to foods containing gluten.^3^ ^,^ ^4^ ^,^ ^5^ In The United States, Kasarda^4^ highlighted the greater intake of wheat and gluten added to whole grain products but found no evidence to support the hypothesis that the genetic improvement of wheat contributed to increasing the number of cases of celiac disease.

According to data from the National Health and Nutrition Examination Survey (2009-2010), the prevalence of celiac disease was 1:141 in the North American population.^6^ In the United Kingdom, the prevalence of the disease was estimated at 1:420 in 2011, and its incidence increased four times between 1990 and 2011 - from 5.2 to 19.1 cases per 100 thousand people/year.^7^ In the city of São Paulo, São Paulo, a sample of four thousand blood donors presented a prevalence of the disease of 1:286.^8^ In Salvador, Bahia, a population-based study conducted with adolescents from public schools identified seroprevalence of 0.49% (6:1,213) for celiac disease.^1^

Health professionals and the media have disseminated information about gluten without scientific basis, leading many people to restrict or exclude foods containing gluten from their diet and assume that some gastrointestinal symptoms are related to the disease. Between 2013 and 2015, the number of people who consumed gluten-free foods increased 67% and sales of these foods rose 136% in the United States.^9^ A gluten-free diet is only recommended for people clinically diagnosed with the disease, given that whole grains are associated with cardiovascular health.^10^ ^,^ ^11^ A cohort study with a 26-year follow-up revealed that gluten is not a risk factor for cardiovascular disease and that its intake was correlated with lower consumption of red meat and total fat and higher consumption of whole grains.^10^

A healthy diet is based on a combination of cereals with other fresh or minimally processed foods, such as beans, vegetables, fruits, meats, and eggs.^12^ Some types of cereals, e.g., wheat, rye, barley, and oat, present two classes of proteins - prolamins and glutenins - in their food matrix that form gluten when combined by manipulation and addition of water.^13^ ^,^ ^14^

Wheat has about 80-85% of its proteins made of gliadin and glutenin, a characteristic that defines it as the greatest source of gluten among cereals.^14^ ^,^ ^15^ Wheat flour is a basic ingredient in the preparation of baking products, to which gluten gives durability^15^ and desired sensory attributes, such as volume and the crunchy and soft texture of baked goods, confectionery, pasta, and others.^14^ However, the food industry widely uses gluten for its technological properties - viscosity, elasticity, moisture, and uniformity.^16^ ^,^ ^17^ Araújo et al.^18^ reported that wheat is commonly added to instant coffee, chocolate powder, ice cream, chewing gum, cold cuts, yogurts, dehydrated soups, tomato sauce, mayonnaise, mustard, among others.

Considering the increasing prevalence of celiac disease and the popularity of gluten-free diets among individuals not diagnosed with the disease, this study aimed to estimate the prevalence of gluten intake according to demographic, socioeconomic, and health-related behavioral variables, as well as identify the main sources of gluten in the diet of adolescents aged 10 to 19 years living in the city of Campinas, São Paulo.

## METHOD

This is a population-based cross-sectional study that included 924 non-institutionalized adolescents (10 to 19 years) living in the urban area of the city of Campinas, São Paulo. We used data from the Health Survey in the City of Campinas (*Inquérito de Saúde no Município de Campinas* - ISACamp 2008-2009), conducted between February 2008 and March 2009.

The survey sample is representative of the population of Campinas and was calculated by probabilistic sampling procedures with a two-stage cluster: census tract and household. In the first stage, 50 census tracts were randomly selected with probability proportional to size (number of households). The second stage selected the households, considering that the total number of adolescents interviewed by tract should not exceed 20.

The sample size was obtained in view of the estimated prevalence of 50%, which corresponds to the maximum variability for the frequency of the events studied, with a confidence level of 95%, sampling error between 4 and 5 percentage points, and design effect of 2, totaling 1,000 individuals aged 10-19 years. Anticipating 20% of refusals and vacant homes, the sample size was adjusted to 1,250. To reach this number, 2,150 households were randomly selected for interviews with adolescents. More details on the sampling process are described on the website http://www.fcm.unicamp.br/fcm/sites/default/files/plano_de_amostragem.pdf.

Information was collected through a questionnaire structured in 14 thematic blocks, including reported morbidities, accidents, and violence; use of health services; preventive practices; use of medicines; health-related behaviors; eating habits; and socioeconomic characteristics. The instrument was previously tested in a pilot study and applied in home interviews by trained and supervised interviewers.

Food intake was estimated by the 24-hour dietary recall (24HR). During the field work, the content of the recalls was checked to identify and solve filling issues. The 24HR was quantified to transform in grams or milliliters the amounts of foods and preparations described in household measures. To that end, we used the information available on tables of household measures,^19^ ^,^ ^20^ food labels, and customer services. Food intake data were entered into the software Nutrition Data System for Research (NDS-R, version 2007, University of Minnesota). Culinary preparations not found on the NDS-R were elaborated based on standardized recipes.^19^ ^,^ ^20^ The software allows the user to include recipes (User Recipe), keeping them separate from the NDS-R database. After being typed, these recipes can be searched by the name given by the user and included in the food directory.

In this study, the dependent variable was gluten intake, created from the encoding of food items mentioned by adolescents in the 24HR. This encoding consisted of recording the foods in an Excel spreadsheet, sorted by the Food Id (food identification number), and assigning codes to these items according to the presence of gluten in them (no=1; yes=2). The diet of these adolescents comprised 565 different foods or preparations, of which 227 contained gluten. To identify the gluten in foods, we searched food labels, websites of food companies, theses and scientific papers related to the topic, and the website of the Brazilian Celiac Foundation (*Associação dos Celíacos do Brasil* - ACELBRA). We included all foods that contained gluten regardless of the amount consumed.

The independent variables selected to analyze factors associated with gluten intake were:

- Demographic and socioeconomic: gender, age group (in years), ethnicity (self-reported) - categorized into white and non-white (black, Asian, multiracial, and indigenous) -, number of people in the household, schooling of the head of the family (in years), monthly per capita household income (according to minimum wage), number of household appliances, whether the adolescent attended school, and place of birth (Campinas, another city in the state of São Paulo, and another state).
- Health-related behaviors: weekly frequency of consumption of fruits, raw and cooked vegetables, milk, beans, and soft drinks, collected through a food frequency questionnaire developed by ISACamp researchers; smoking (percentage of adolescents who smoked, regardless of the frequency and intensity of cigarette use); alcohol consumption classified into “does not drink” and “drinks” (from one to four times per month or two or more times per week); time (hours/day) spent watching TV and using the computer; and physical activity in leisure time, obtained by the frequency (number of days per week) and duration (minutes per day) of exercises, such as walking, running, gymnastics, weight training, dancing, swimming, cycling, and playing soccer, volleyball, basketball, among others. Adolescents aged 10-17 years who practiced physical activity for at least 60 minutes per day, five or more days a week, and those aged 18-19 years who practiced at least 150 minutes per week, distributed into at least three days, were considered active.^21^

Data analysis revealed an association between independent variables and gluten intake, through the chi-square test, with a significance level of 5%. We estimated prevalence ratios (PR) and their respective confidence intervals of 95% (95%CI) using simple Poisson regression. Next, we developed a multiple Poisson regression model in two stages. The first stage consisted of entering all demographic and socioeconomic variables with p<0.20 in the bivariate analysis and those with p<0.05 remaining in the model. The second stage added to the model health-related behavioral variables with p<0.20 in the bivariate analysis, keeping those with p<0.05. The model was adjusted for dietary energy (kcal), following the recommendation from Willett et al.^22^

We performed statistical analyses using the software Stata 11.0 (Stata Corp., Chicago, USA) in the svy module, which considers the weights and complex sampling design of the study.

The Research Ethics Committee of Universidade Estadual de Campinas (UNICAMP) approved the project ISACamp 2008-2009, under Report No. 079/2007. Parents or guardians of adolescents younger than 18 years had to sign an informed consent form.

## RESULTS

The study included 924 adolescents, aged 10 to 19 years, who filled a 24HR. The mean age of the population surveyed was 14.1 years (95%CI 13.9-14.4) and 51% of them were females.

The estimated prevalence of gluten intake reached 26.9% and was significantly higher in adolescents of better socioeconomic status, characterized by higher strata of schooling of the head of the family, household income, number of household appliances, and attending a private school. On the other hand, we found lower prevalence in participants aged 15-19 years (at the threshold of statistical significance), individuals who declared being non-white, and those born in other states (Table 1).

**Table 1:** Prevalence and prevalence ratio of gluten intake, according to demographic and socioeconomic variables in adolescents aged 10-19 years. Health Survey in the City of Campinas (*Inquérito de Saúde no Município de Campinas* - ISACamp), 2008-2009.

	n	% (95%CI)	p-value*	PR (95%CI)
Gender				
Male	466	26.7 (25.1-28.4)	0.582	1
Female	458	27.2 (25.2-29.4)		1.02 (0.95-1.09)
Total	924	26.9 (25.3-28.6)		
Age group (years)				
10 to 14	508	27.8 (26.0-29.7)	0.050	1
15 to 19	416	25.9 (24.0-27.9)		0.93 (0.86-1.00)
Ethnicity (self-reported)				
White	593	27.8 (26.3-29.3)	0.032	1
Non-white	328	25.4 (23.1-27.8)		0.91 (0.84-0.99)
Place of birth				
Campinas	701	27.8 (26.1-29.5)	0.010	1
Another city in the state of São Paulo	104	25.5 (22.8-28.4)		0.92 (0.81-1.04)
Another state	119	23.6 (21.1-26.2)		0.85 (0.76-0.94)
Number of people in the household				
1 to 2	66	28.6 (25.2-32.3)	0.116	1
3 to 4	443	27.7 (25.9-29.5)		0.97 (0.86-1.08)
5 or +	415	25.8 (23.8-27.9)		0.90 (0.78-1.04)
Schooling of the head of the family (years)				
0 to 7	387	24.2 (22.3-26.2)	<0.001	1
8 to 11	313	26.9 (25.0-28.8)		1.11 (1.02-1.21)
12 or +	213	30.9 (28.6-33.3)		1.27 (1.14-1.42)
Per capita household income (according to minimum wage**)				
< 1	585	25.6 (24.0-27.4)	0.001	1
≥1 and ≤ 3	264	28.4 (26.1-30.8)		1.11 (1.01-1.20)
> 3	75	31.1 (28.5-33.9)		1.21 (1.09-1.35)
Number of household appliances				
0 to 7	190	22.8 (20.2-25.7)	<0.001	1
8 to 15	468	26.5 (24.9-28.3)		1.16 (1.04-1.29)
16 or +	265	30.2 (28.4-32.1)		1.32 (1.17-1.49)
Attends school				
No	144	24.2 (21.6-27.0)	<0.001	1
Yes, public school	617	26.3 (24.5-28.2)		1.09 (0.98-1.20)
Yes, private school	162	31.2 (28.8-33.7)		1.29 (1.13-1.46)

n: number of individuals in the unweighted sample; 95%CI: confidence interval of 95%; *chi-square test; **minimum wage at the time of the survey: January to April/2008 = R$ 415 and May/2008 to April/2009 = R$ 450.

Table 2 indicates a higher prevalence of gluten intake among adolescents who consumed fewer beans and vegetables during the week, as well as those who used the computer.

**Table 2:** Prevalence and prevalence ratio of gluten intake, according to health-related behavioral variables in adolescents aged 10-19 years. Health Survey in the City of Campinas (*Inquérito de Saúde no Município de Campinas* - ISACamp), 2008-2009.

	n	% (95%CI)	p-value*	PR (95%CI)
Fruits (times a week)				
≥4	439	27.1 (25.4-28.9)	0.778	1
<4	485	26.8 (24.4-29.2)		0.98 (0.89-1.09)
Raw vegetables (times a week)				
≥4	517	25.6 (23.8-27.4)	0.004	1
<4	407	28.9 (26.7-31.1)		1.13 (1.04-1.22)
Cooked vegetables (times a week)				
≥4	345	25.2 (23.5-27.0)	0.004	1
<4	579	28.1 (26.1-30.1)		1.11 (1.03-1.19)
Milk (times a week)				
≥4	650	26.9 (25.1-28.7)	0.792	1
<4	274	27.2 (24.8-29.7)		1.01 (0.92-1.10)
Beans (times a week)				
≥4	767	25.9 (24.4-27.5)	<0.001	1
<4	157	32.8 (30.0-35.7)		1.27 (1.16-1.38)
Soft drink (times a week)				
≥4	359	27.5 (25.6-29.4)	0.343	1.03 (0.96-1.10)
<4	565	26.6 (24.8-28.5)		1
Smoking				
Never smoked	882	26.9 (25.4-28.6)	0.894	1
Ex-smoker/smoker	42	27.2 (22.6-32.4)		1.01 (0.86-1.19)
Alcohol consumption				
Does not drink	773	26.7 (25.1-28.5)	0.306	1
Drinks	146	28.1 (25.4-31.0)		1.05 (0.95-1.16)
Physical activity in leisure time				
Active	197	25.7 (23.0-28.5)	0.232	1
Inactive or insufficiently active	727	27.3 (25.7-29.0)		1.06 (0.97-1.18)
Time spent watching TV (hours/day)				
<3	370	27.3 (25.5-29.2)	0.481	1
≥3	540	26.6 (24.7-28.6)		0.97 (0.90-1.05)
Computer use (hours/day)				
0	442	24.9 (22.8-27.1)	<0.001	1
1 to 3	369	28.3 (26.5-30.1)		1.13 (1.04-1.23)
4 or +	107	30.4 (28.2-32.7)		1.22 (1.09-1.36)

n: number of individuals in the unweighted sample; 95%CI: confidence interval of 95%; *chi-square test; PR: prevalence ratio.

Table 3 presents the results of the hierarchical multiple Poisson regression model. Gluten intake proved to be lower in adolescents aged 15-19 years and higher in participants who lived in households headed by individuals with 12 or more years of schooling, attended school, had eight or more household appliances, and consumed beans and raw vegetables less than four times per week.

**Table 3:** Poisson multivariate regression model. Health Survey in the City of Campinas (*Inquérito de Saúde no Município de Campinas* - ISACamp), 2008-2009.

	First stage PR_adjusted_* (95%CI)	Second stage PR_adjusted_** (95%CI)
Age group (years)		
10 to 14	1	1
15 to 19	0.92 (0.85-0.98)	0.92 (0.86-0.98)
Schooling of the head of the family (years)		
0 to 7	1	1
8 to 11	1.08 (0.99-1.18)	1.08 (0.99-1.17)
12 or +	1.15 (1.03-1.28)	1.15 (1.03-1.28)
Attends school		
No	1	1
Yes, public school	1.11 (1.00-1.22)	1.10 (1.00-1.21)
Yes, private school	1.18 (1.05-1.34)	1.16 (1.02-1.31)
Number of household appliances		
0 to 7	1	1
8 to 15	1.12 (1.01-1.23)	1.11 (1.01-1.22)
16 or +	1.21 (1.08-1.34)	1.20 (1.07-1.32)
Leafy vegetables (times a week)		
≥4		1
<4		1.10 (1.03-1.16)
Beans (times a week)		
≥4		1
<4		1.16 (1.07-1.26)

PR: prevalence ratio; *PR adjusted for energy (kcal) and demographic and socioeconomic variables; **PR adjusted for energy (kcal) and all variables in the table; 95%CI: confidence interval of 95%.

**Figure 1:**
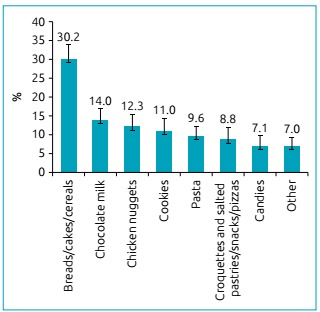
Types and confidence interval of 95% of foods containing gluten in the diet of adolescents. Health Survey in the City of Campinas (*Inquérito de Saúde no Município de Campinas* - ISACamp), 2008-2009.

Regarding food sources of gluten, bread, cakes, and cereals (oat, wheat bran, granola, corn flakes, and cereal flour) represented 30.2% (95%CI 28.4-32.1) of the diet of adolescents; chocolate milk, 14% (95%CI 12.5-15.5); chicken nuggets, 12.3% (95%CI 10.6-13.9), cookies, 11% (95%CI 9.3-12.7); pasta, 9.6% (95%CI 8.3-10.9); croquettes and salted pastries, snacks, and pizzas, 8.8% (95%CI 7.2-10.4); candies, 7.1% (95%CI 5.7-8.4); and other foods such as packaged snacks and processed sauces, 7% (95%CI 5.9-8.2) (Figure 1).

## DISCUSSION

The results of this study show higher prevalence of gluten intake among younger adolescents (10 to 14 years) and subgroups with better socioeconomic status, assessed by the level of education of the head of the family and number of household appliances owned, those who attended school, and consumed less beans and leafy vegetables during the week.

In the National Food Survey (*Inquérito Nacional de Alimentação* - INA 2008-2009), adolescents (10-19 years) showed high percentages of food intake outside the home in all Brazilian regions compared to adults and older adults, especially cakes and cookies (20.9%), snacks and crackers (25.9%), candies (36.2%), pizza (37.5%), sandwiches (40.5%), and croquettes and salted pastries (51.9%).^23^ In São Paulo, Andrade et al.^24^ evaluated adolescents aged 12-19 years and found a significant reduction in diet quality after they turned 16. Adolescence is marked by social and behavioral changes that negatively affect food choices.^25^ Nonetheless, with increasing age, the prevalence of gluten intake decreases in this population, which can be justified by the drop in consumption of chocolate milk, from 15.8% (95%CI 14.0-17.6) to 11.5% (95%CI 9.3-13.6) in the age groups 10-14 and 15-19 years, respectively (data not shown in table), a plausible explanation considering the substitution of milk for sugary drinks.^26^ ^,^ ^27^

Socioeconomic status was associated with a higher prevalence of gluten intake, a result observed among those who owned more household appliances and lived in households headed by better-educated people. Data from the National Adolescent Student Health Survey (*Pesquisa Nacional de Saúde do Escolar* - PeNSE 2009) revealed a decreasing trend in the consumption of beans and an increasing one in the intake of candies, cookies, and cold cuts with the improvement in goods and services score (having a TV, refrigerator, stove, washing machine, among others, and a domestic worker in the household).^28^ Better income and education levels of the head of the family contribute to improving dietary variety and consumption of healthy foods, e.g., fruits, vegetables, and milk;^29^ ^,^ ^30^ but they also provide more access to food items such as processed meats, cookies, pies, packaged snacks, candies, pizzas, and ready-made meals.^31^

This study found an association between being enrolled in school, regardless of the administrative affiliation, and greater gluten intake. In Brazil, all public school students benefit from the National School Feeding Program (*Programa Nacional de Alimentação Escolar* - PNAE), which has to meet their nutritional needs during school hours.^32^ The higher gluten intake observed in adolescents from public schools compared to those who do not attend school can be probably explained by the school menu including formulated foods (dry pre-mixes), cookies, bread, cakes, granola bars, among others.^33^ ^,^ ^34^ According to PeNSE 2012, cafeterias were more common in private schools (94.8%) than public ones (39.4%), but an alternative point of sale was available for 44.8 and 33.3% of students from public and private schools, respectively.^35^ In cafeterias, the most frequent food items were salted pastries (39.4%) and ice cream, chocolate, and candies (32%), while in points of sale, they were candies (33.2%), croquettes (29.6%), and packaged snacks (29.1%).^35^

The low frequency of consumption of beans and leafy vegetables was associated with a higher prevalence of gluten intake. National data from 1987 to 2009 indicated a decreasing trend in the household acquisition of foods such as rice, beans, milk, vegetables, roots, and tubers.^31^ Comparing the results of PeNSE 2009 and 2012, Malta et al.^36^ found a reduction in the consumption of beans (from 62.5 to 60.0%) and fruits (31.5 to 29.8%) among students. Another relevant issue is the substitution of main meals (lunch and dinner) for snacks, which reaches 16.2% (95%CI 15.5-16.8) in the adult population (≥18 years) living in Brazilian state capitals and the Federal District.^37^ Teixeira et al.^38^ revealed that 51.4% and 34.0% of adolescents from São Paulo exchanged dinner and lunch, respectively, for snacks, including sandwiches with and without hamburger, croquettes, baked pastries, hot dogs, and pizza.

In this study, the most common foods containing gluten in the diet of the individuals assessed were bread/cakes and cereals, chocolate milk, chicken nuggets, and cookies. INA ­2008-2009 also identified some of these food groups. The 20 foods most consumed by adolescents included bread (60.9%), pasta (19.0%), croquettes and salted pastries (17%), crackers (15.8%), cakes (13.4%), and cookies (12.7%).^39^

Data analysis of this study should consider that the application of a single 24HR does not portray the usual intake of an individual, due to the wide intra- and interpersonal variation in food consumption.^40^ Nevertheless, if the 24HR is population-based and takes into account the different days of the week and months of the year, it is possible to estimate the mean intake for the population evaluated.^41^ Also, the prevalence of gluten intake might be overestimated, as few recalls included food brands, which would allow us to check the information. Also, we emphasize that ISACamp did not intend to investigate gluten-related diseases. Regarding the task of encoding food items, the main difficulties found were the fact that not all company websites displayed information about the presence of gluten in their products, the multiplicity of brands for a single item, and the undetailed content on the ACELBRA website.

Gluten intake was associated with lower consumption of beans and vegetables, indicating the adoption of a worse dietary pattern. Adolescents with higher socioeconomic status were more exposed to gluten. Given the increasing prevalence of gluten-related diseases, the changes in eating habits, and the popularity of gluten-free diets, we suggest the development of food education strategies to promote healthy dietary choices and inform adolescents about the dangers of fad diets.
